# Activation of the miR-34a/SIRT1/p53 Signaling Pathway Contributes to the Progress of Liver Fibrosis *via* Inducing Apoptosis in Hepatocytes but Not in HSCs

**DOI:** 10.1371/journal.pone.0158657

**Published:** 2016-07-07

**Authors:** Xiao-Feng Tian, Fu-Jian Ji, Hong-Liang Zang, Hong Cao

**Affiliations:** Department of General Surgery, the China-Japan Union Hospital, Jilin University, Changchun 130021, China; University of Alabama at Birmingham, UNITED STATES

## Abstract

Liver fibrosis results from a sustained wound healing response to chronic liver injury, and the activation of nonparenchymal hepatic stellate cells (HSCs) is the pivotal process. MicroRNA-34a (miR-34a) is the direct target gene of p53 and activates p53 through sirtuin 1 (SIRT1) simultaneously. The miR-34a/SIRT1/p53 signaling pathway thus forms a positive feedback loop wherein p53 induces miR-34a and miR-34a activates p53 by inhibiting SIRT1, playing an important role in cell proliferation and apoptosis. miR-34a expression has been found to be increased in animal models or in human patients with different liver diseases, including liver fibrosis. However, the exact role of this classical miR-34a/SIRT1/p53 signaling pathway in liver fibrosis remains unclear. In the present study, using a CCl_4_-induced rat liver fibrosis model, we found that the miR-34a/SIRT1/p53 signaling pathway was activated and could be inhibited by SIRT1 activator SRT1720. Further studies showed that the miR-34a/SIRT1/p53 signaling pathway was activated in hepatocytes but not in HSCs. The activation of this pathway in hepatocytes resulted in the apoptosis of hepatocytes and thus activated HSCs. Our data indicate that the miR-34a/SIRT1/p53 signaling pathway might be a promising therapeutic target for liver fibrosis.

## Introduction

Liver fibrosis, characterized with the excessive accumulation of extracellular matrix (ECM) proteins, results from a sustained wound-healing response to chronic liver injury including chronic viral hepatitis, alcohol-induced damage, non-alcoholic steatohepatitis and autoimmune liver disease [1, 2]. Liver fibrosis may progress to liver cirrhosis or hepatocellular carcinoma, which is associated with significant morbidity and mortality [1, 3, 4]. Traditionally, liver fibrosis was considered irreversible; however, mounting evidence indicates that even advanced fibrosis is reversible [2, 3]. The pathogenesis of liver fibrosis is complex and varies in different liver injuries. Following chronic liver injury, liver parenchymal cell hepatocytes generally undergo apoptosis, leading to the activation of non-parenchymal hepatic stellate cells (HSCs), which are the primary source of ECM and responsible for scar formation [3]. The activation of HSCs is the pivotal process in liver fibrosis [2]. Targeting apoptosis, including the induction of HSC apoptosis and the protection of hepatocytes from apoptosis, is one important method to attenuate liver fibrosis [3].

MicroRNAs (miRNAs), a group of small non-coding RNA molecules, modulate the expression of many genes by binding to the complementary sequences of the 3’ untranslated region in the targeted mRNA [5, 6]. miRNAs play an important role in the development, physiology and pathophysiology of animals and plants by regulating the proliferation, differentiation and apoptosis of cells [5, 7, 8]. The misregulation of miRNAs is involved in many human organ disorders, including liver diseases [6, 9–11]. Many miRNAs have been identified as being involved in the process of non-alcoholic steatohepatitis (NASH), liver fibrosis and hepatocellular carcinoma (HCC) [12–14].

miR-34a expression has been found to be increased in animal models or human patients with alcoholic liver injury, non-alcoholic fatty liver disease (NAFLD), liver fibrosis or HCC, and its expression level correlates with disease severity [15–20]. miR-34a is the direct target gene of p53, and one of the miR-34a targets is sirtuin 1 (SIRT1), which can inhibit p53-dependent apoptosis by deacetylating all major p53 acetylation sites [21, 22]. Thus, the miR-34a/SIRT1/p53 signaling pathway forms a positive feedback loop wherein p53 induces miR-34a and miR-34a then activates p53 by inhibiting SIRT1, playing an important role in cell proliferation and apoptosis [22]. The miR-34a/SIRT1/p53 signaling pathway was reported to be activated in NAFLD and involved in the apoptosis of hepatocytes [16]. Because miR-34a is also upregulated in liver fibrosis, the extent to which the miR-34a/SIRT1/p53 signaling pathway is involved in liver fibrosis and the manner in which it affects hepatocytes and HSCs are unclear. The present study aimed to investigate the functions of miR-34a in CCl₄-induced liver fibrosis in rats.

## Materials and Methods

### Cell lines

Human normal liver L-02 cells and human HSC lines LX-2 were obtained from Type Culture Collection of Chinese Academy of Sciences (Shanghai, China) and cultured in RPIM1640 (Invitrogen, Carlsbad, CA, USA) supplemented with 10% fetal bovine serum (FBS; Gibco BRL, Grand Island, NY, USA) and antibiotics (100 units/mL penicillin and 100 μg/mL streptomycin) at 37°C with 5% CO_2_.

### Animals and liver fibrosis model

Male Sprague Dawley rats (weighing 200–250 g) were obtained from the animal center of Jilin University. They were maintained at 25°C on a 12 h light/dark cycle and were allowed free access to chow and water during the experimental period. The experimental procedures were approved by the Ethics Committee on Animal Care of the China-Japan Union Hospital of Jilin University (No. 20130923). Using randomized computer-generated numbers, rats were randomly allocated to four groups (n = 24 in each group): a control group, a SRT1720 (SIRT1 activator; Selleck, Houston, TX, USA) -treated group, a CCl_4_-treated group, and a CCl_4_ + SRT1720 group. Liver fibrosis rats were established through intraperitoneally injecting with 0.2 mL/kg of CCl_4_ (mixed in 1:1 vegetable oil) twice a week for 4 weeks and then once a week for the next 12 weeks. For the CCl_4_ + SRT1720 group, in addition to CCl_4_, rats were treated every other day with an intraperitoneal injection of SRT1720 at doses of 50 mg/kg. For SRT1720-treated group, rats were treated as rats in the CCl_4_ + SRT1720 group without CCl_4_ treatment.

Samples were collected at 4, 8 and 12 weeks (n = 8 at each time). The animals were anesthetized with sodium pentobarbital (100 mg/kg), and blood samples were collected for serum biochemistry. Three rats were then sacrificed by cervical dislocation, and the livers were then quickly removed and divided into three parts. One part was fixed in paraformaldehyde and paraffin-embedded for histological assessment, another part was used for Western blot, and the third part was used for RNA extraction. Another five rats were used for the isolation of primary cells.

### Histopathology

Paraffin-embedded tissue was cut into 5-μm-thick slides and stained with hematoxylin and eosin (H&E). The morphological changes in the samples were observed under a light microscope.

### Enzyme-linked immunosorbent assay ELISA

Serum levels of alanine aminotransferase (ALT), aspartate aminotransferase (AST), tumor necrosis factor (TNF)-α and tumor growth factor (TGF)-β were determined using ELISA according to the manufacturer’s protocols. The ELISA kit for ALT, AST, TNF-α and TGF-β was obtained from Shanghai Kemin bioscience Ltd., China.

### Isolation of primary hepatocytes, nonparenchymal cells and HSCs

Primary hepatocytes and nonparenchymal cells were isolated from rats using a standard in situ 2-step collagenase perfusion procedure as described previously [23]. Hepatocytes were isolated from nonparenchymal cells by subsequent centrifugation at 50 g for 3 min, and nonparenchymal cells in the supernatant were pelleted at 600 g for 10 min. HSCs were isolated from nonparenchymal cells using Nycodenz separation liquid for centrifugation at 1500 g for 15 min. Both hepatocytes and HSCs were applied for Western blot.

### RNA extraction and quantitative RT-PCR (RT-qPCR)

Total RNA was extracted from livers or cells using TRIzol Reagent (Invitrogen, Carlsbad, CA) according to the manufacturer’s protocol.

For miR-34a detection, first-strand cDNA was synthesized using the Taqman miRNA RT Kit (Applied Biosystems, Carlsbad, CA, USA). For detection of the miRNA level by RT-qPCR, a TaqMan^@^ microRNA assay (Applied Biosystems) was used to quantify the relative expression level of miR-34a (assay ID. 000426), with U6 used (assay ID. 001973) as an internal control, in an Applied Biosystems 7500 Detection system (Applied Biosystems). The relative amount of miR-34a was normalized against U6 snRNA, and the fold change for each miRNA was calculated by the 2-^ΔΔCt^ method [24]. The relative miR-34a expression was calculated from three different experiments.

For mRNA detection, RT-qPCR was performed using a One Step SYBR ® PrimeScript ® PLUS RT-PCR Kit (Takara, Shiga, Japan) according to the manufacturer’s protocol. The primers used in this study are presented in Table 1.

**Table 1:** Primers used in the present study.

Gene	Forward sequence (5'-3')	Reverse sequence (5'-3')
α-SMA	GGCTCTGGGCTCTGTAAGG	CTCTTGCTCTGGGCTTCATC
collagen I	AACATGACCAAAAACCAAAAGTG	CATTGTTTCCTGTGTCTTCTGG
TGF-β1	GGCAGTGGTTGAGCCGTGGA	TGTTGGACAGCTGCTCCACCT
GAPDH	GCACCGTCAAGGCTGAGAAC	TGGTGAAGACGCCAGTGGA

### Co-culture of hepatocytes and HSCs

First the effect of miR-34a on hepatocytes were analyzed. Hepatocyte L-02 cells were seeded 6-well plates 4 h before transfection, and then transfected with mimics of miR-34a or negative control miRNAs (Jima, Shanghai, China) using Lipofectamine RNAiMAX Transfection Reagent (Invitrogen). After 6 h incubation with transfection mix, cells were maintained in DMEM with 10% FBS, with or without 20 μg/mL SRT1720. The expression of miR-34a in hepatocytes was determined 24 h after transfection. Forty-eight hours later, the cells were harvested for Western blot and apoptosis assay.

Next the impact of miR-34a transfected hepatocytes on the functional maturation of HSCs was investigated using a co-culture system. Briefly, hepatocyte L-02 cells were seeded into the bottom chamber of rat-tail collagen-coated 6-well transwell plates (0.4-μm-pore membrane, Thermo Fisher Scientific) 4 h before transfection, and then transfected with mimics of miR-34a or negative control miRNAs with or without 20 μg/mL SRT1720. Twenty-four hours later, HSCs LX-2 cells were seeded at 10^5^ cells/well in the top chamber to co-culture for another 24 h without SRT1720. HSCs in the top chamber were harvested for Western blot.

### Western blot

Homogenized tissue or harvested cells in a commercial lysis buffer (Beyotime, Zhejiang, China) were first quantified using the BCA method (Pierce; Rockford, IL, USA), and then denatured by boiling for 5 min. Forty micrograms of protein per sample was denatured, separated by SDS-PAGE (SDS-polyacrylamide gel electrophoresis) and transferred to a polyvinylidene difluoride membrane (Millipore, Billerica, MA, USA). The membranes were blocked with Tris-buffered saline (TBS) containing 0.1% Tween-20 (TBST) and 5% (w/v) nonfat dry milk for 1 h. The membranes were then incubated overnight at 4°C with the primary antibodies of α-smooth muscle actin (SMA), SIRT1, p53, acetylated p53 (Ac-p53), collagen I, caspase3, and β-actin (Abcam, Cambridge, MA) at a dilution of 1:1000. After washing with TBST, the membranes were incubated with horseradish peroxidase-conjugated secondary antibodies (Sangon, China) at a dilution of 1:2000 at room temperature for 1 h. The signals were visualized using an electrochemiluminescence kit (Pierce Biotechnology, Rockford, IL, USA) following the manufacturer’s instructions and captured digitally using chemiluminescence imaging systems (Shanghai Clinx Science Instruments, China). The bands were quantitated using ImageJ software.

### Apoptosis assay

The apoptosis of hepatocytes was determined using an Annexin V-FITC/PI Apoptosis Detection Kit (BD Bioscience, Franklin Lakes, NJ, US) according to the manufacturer’s protocol. Flow cytometry analysis was performed with a fluorescent-assisted cell sorting (FACS) machine using FlowJo software (BD FACSCalibur).

### Statistical analysis

The data are expressed as the means ± SD. The data were analyzed using SPSS 20 (SPSS, Chicago, IL, USA). Group comparisons were conducted using analysis of variance with a Tukey post-hoc test. A significant difference was considered at *P* < 0.05.

## Results

### SIRT1 is involved in liver fibrosis

**Fig 1:**
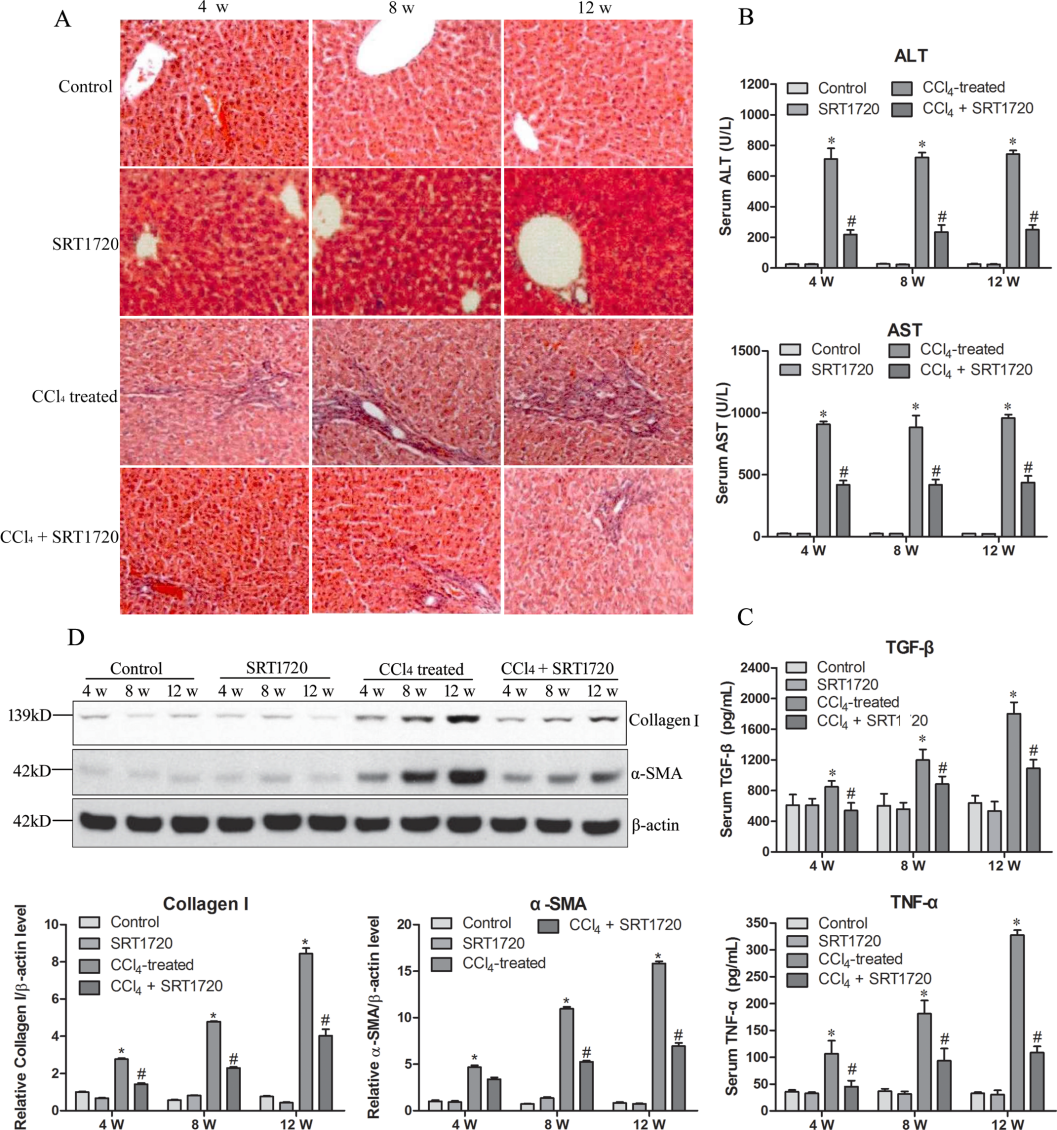
Upregulation of SIRT1 inhibits liver fibrosis. Rats were divided into four groups: Control, SIRT1 activator SRT1720-treated group, liver fibrosis model (CCl_4_ treated) and SRT1720-treated liver fibrosis model (CCl_4_ + SRT1720). The liver fibrosis model was established through intraperitoneally injecting rats with 0.2 mL/kg of CCl_4_ (mixed in 1:1 vegetable oil) twice a week for 4 weeks and then once a week for the next 12 weeks. For the CCl_4_ + SRT1720 group, in addition to CCl_4_, rats were treated every other day with an intraperitoneal injection of SRT1720 at doses of 50 mg/kg. Rats were sacrificed at 4, 8 and 12 w. The liver fibrosis of the model was confirmed by hematoxylin and eosin staining, showing the histopathology of liver (A), serum levels of alanine aminotransferase (ALT), aspartate aminotransferase (AST) (B), tumor necrosis factor (TNF)-α and tumor growth factor (TGF)-β (C) showing the liver function and immunology response, and expression of α-SMA (D). * *P*<0.05 compared to control. # *P*<0.05 compared to CCl_4_-treated group.

To investigate the expression of miR-34a/SIRT/p53 signaling proteins and the possible function of this signaling pathway during liver fibrosis *in vivo*, we established a CCl_4_-induced rat liver fibrosis model and studied the effect of the SIRT1 activator SRT1720 on liver fibrosis. Histopathology changes and serum levels of ALT and AST were determined in liver fibrosis rats with or without SRT1720 treatment. H&E staining revealed that the liver fibrosis progressed more in the CCl_4_-treated rats compared to control rats and only SRT1720-treated rats, while SRT1720 treatment alleviated liver fibrosis in CCl_4_-treated rats (Fig 1A). Similarly, CCl_4-_induced rats had significantly higher levels of ALT and AST compared to controls or only SRT1720-treated rats, while SRT1720 treatment decreased the levels of ALT and AST in the CCl_4_-induced rats (Fig 1B). Serum levels of the inflammatory factors TNF-α and TGF-β, as well as the expression of the main liver fibrosis marker α-SMA, were also examined. The serum levels of TNF-α and TGF-βwere dramatically higher in the CCl_4_-induced rats, but SRT1720 treatment significantly inhibited the CCl_4_-induced increase in TNF-α and TGF-β expression (Fig 1C). The expression of α-SMA and collagen I showed similar changes (Fig 1D). These data suggest that the liver fibrosis rat model was established and that SIRT1 displayed an anti-fibrotic effect during the progression of liver fibrosis.

### The miR-34a/SIRT1/p53 signaling pathway is activated during liver fibrosis

**Fig 2:**
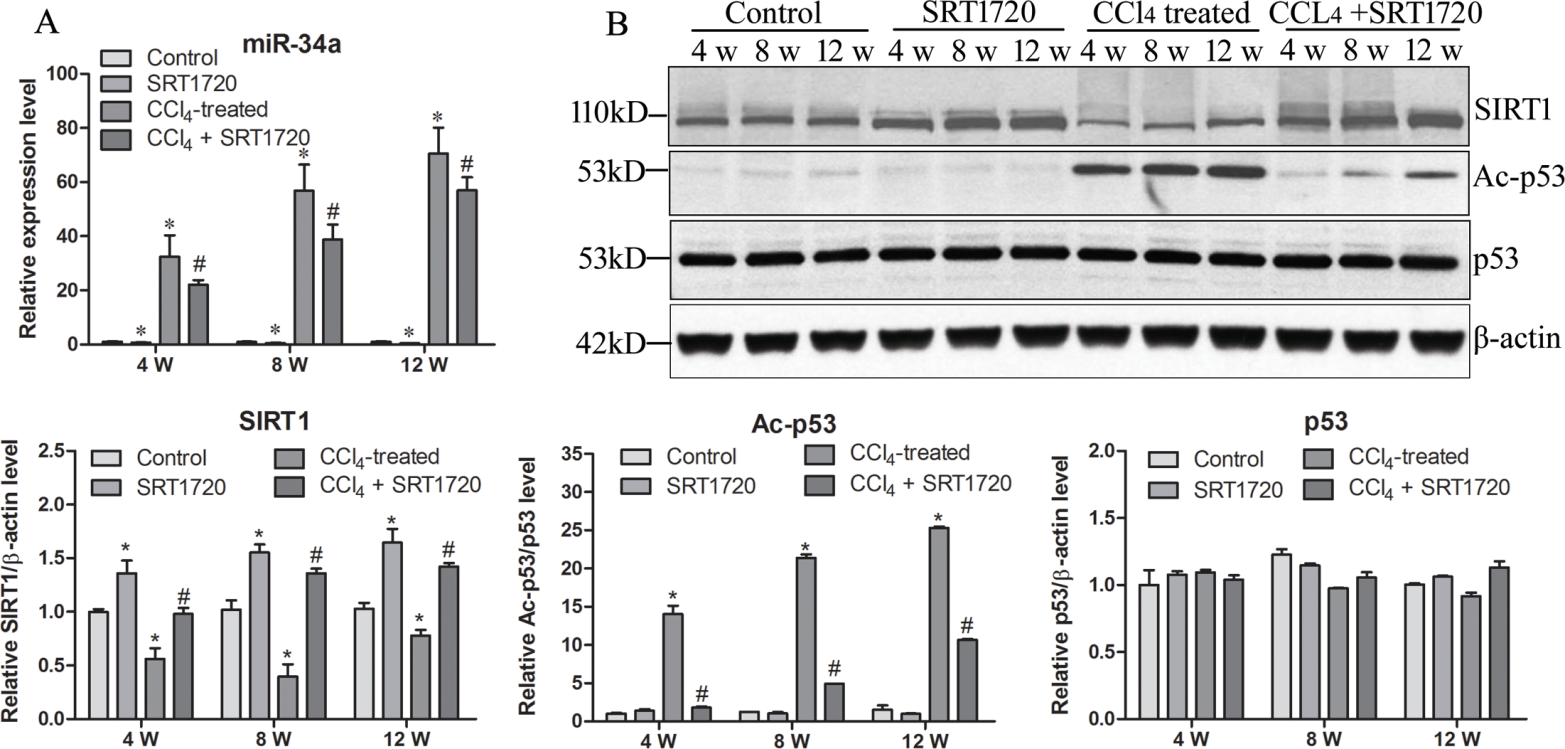
miR-34a/SIRT1/p53 signaling pathway is activated during liver fibrosis. Expression of miR-34a (A), SIRT1, p53, acylated p53 (Ac-p53) and β-actin (B) were detected in four rat groups at three time points. * *P*<0.05 compared to control. # *P*<0.05 compared to CCl_4_-treated group.

Next, the expression of miR-34a, SIRT1, p53 and Ac-p53 were determined. As shown in Fig 2A, miR-34a expression increased significantly in a CCl_4_ treatment time-dependent manner, and SRT1720 treatment significantly reversed the CCl_4_-induced upregulation of miR-34a. Subsequently, SIRT1 expression decreased in the CCl_4_-induced liver fibrosis rats, but SRT1720 treatment reversed this downregulation (Fig 2B). As a result, p53 acetylation was increased without altering total p53 expression in CCl_4_-induced liver fibrosis rats, and SRT1720 treatment reversed this change, leading to a decrease in acetylated p53 levels (Fig 2B). These data indicate that the miR-34a/SIRT1/p53 signaling pathway was activated in the liver fibrosis process.

### The miR-34a/SIRT1/p53 signaling pathway is activated in hepatocytes but not in HSCs

**Fig 3:**
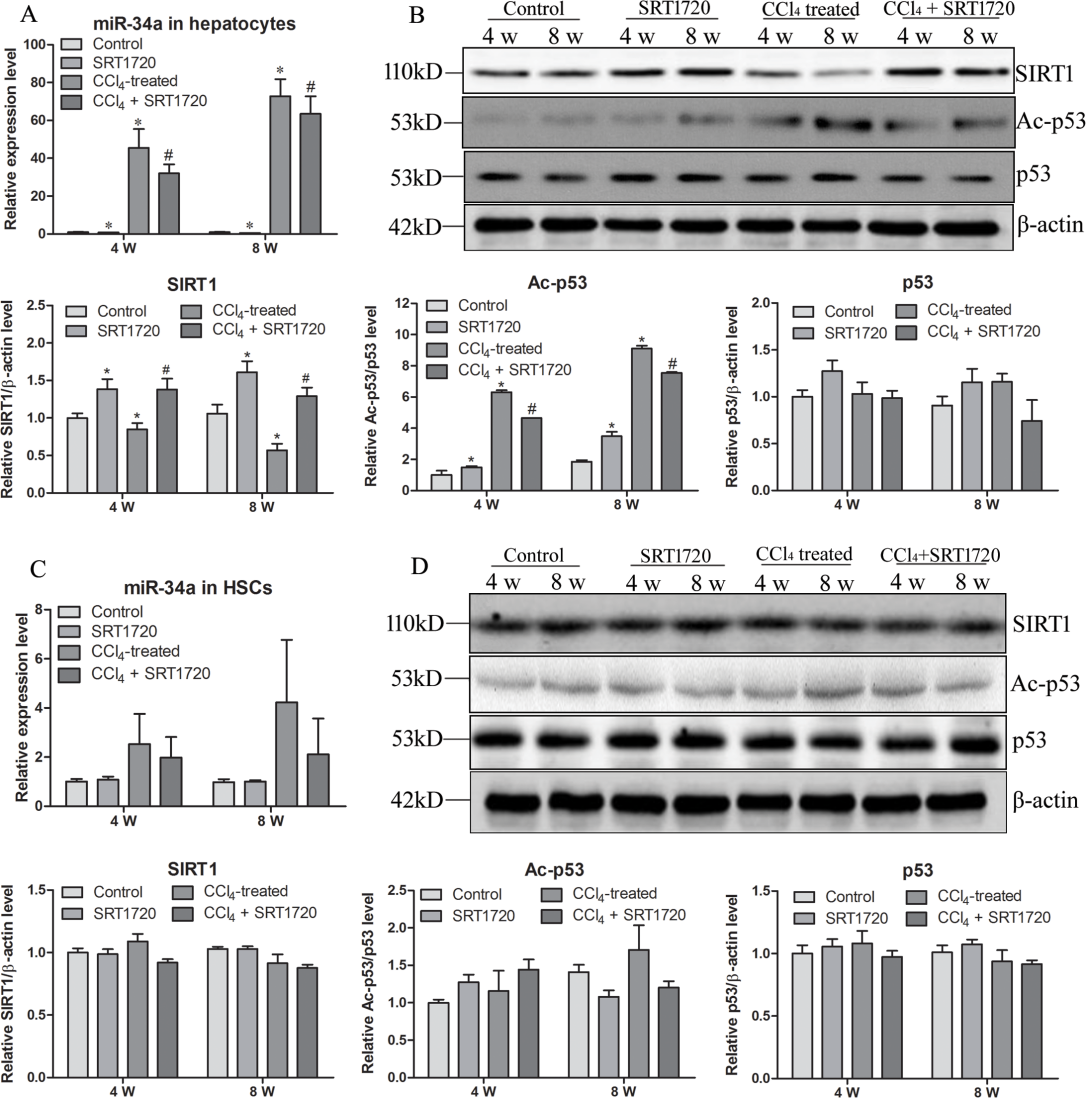
miR-34a/SIRT1/p53 signaling pathway is activated in hepatocytes but not in HSCs. Primary hepatocytes and HSCs were isolated from rats at 4 and 8 w. Expression of miR-34a (A, C), SIRT1, p53, Ac-p53 and β-actin (B, D) were detected in the two types of primary cells. A, B, primary hepatocytes. C, D, primary HSCs. * *P*<0.05 compared to control. # *P*<0.05 compared to CCl_4_-treated group.

To further investigate the underlying mechanism of the miR-34a/SIRT1/p53 signaling involved in liver fibrosis, primary hepatocytes and HSCs were isolated in four rat groups, and the expression of miR-34a, SIRT1, p53 and Ac-p53 in hepatocytes and HSCs were analyzed. Because of the serious fibrosis of the liver in the CCl_4_-treated rats at 12 w, primary hepatocytes isolation was unsuccessful. Thus, only samples from 4 and 8 weeks were applied for analysis. The results in the hepatocytes were similar to those in the rat liver. miR-34a was upregulated in the CCl_4_ group and showed slightly decreased expression in the CCl_4_ + SRT1720-treated group (Fig 3A). SIRT1 was downregulated in the CCl_4_ group, and SRT1720 treatment reversed this downregulation (Fig 3B). In addition, p53 acetylation was higher in the CCl_4_ group but lower in the CCl_4_ + SRT1720-treated group, without altering the total p53 expression (Fig 3B). However, no changes in the expression of miR-34a or these proteins were observed in primary HSCs (Fig 3C and 3D), although the variability of miR-34a in primary HSCs was a little great, which may be related with the culture activation of primary HSCs isolated from different rats.

### The miR-34a-induced apoptosis of hepatocytes activates HSCs

**Fig 4:**
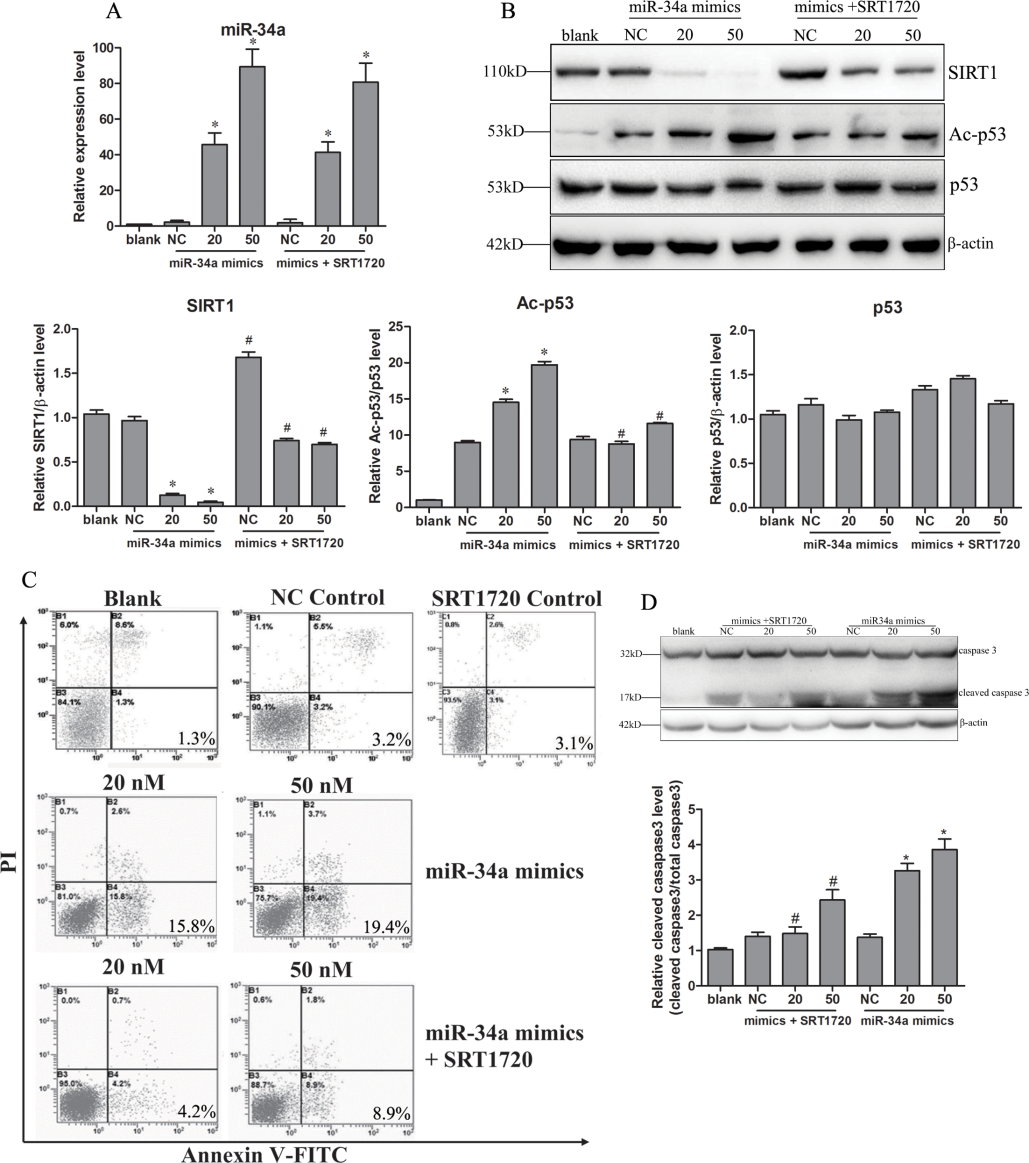
Upregulation of miR-34a-induced apoptosis of hepatocytes. Human normal hepatocytes cell line L-02 cells were transfected with miR-34a mimics (20, 50 nM) or negative miRNA control (NC) with or without SRT1720 for 48 h. Expression of miR-34a was confirmed (A) at 24 h after transfection. Changes in the expression of SIRT1, p53 and Ac-p53 were determined (B) after 48 h. The apoptosis of hepatocytes was detected using an Annexin V-FITC/PI Apoptosis Detection Kit in a flow cytometry system (C) or by detecting the cleavage of caspase3 (D). Blank, L-02 cells; NC, negative control (L-02 cells transfected with negative mimics). * *P*<0.05 compared to NC. # *P*<0.05 compared to mimics transfection.

Because miR-34a upregulation was only observed in hepatocytes, we wondered how miR-34 functions in liver fibrosis. Thus, we examined the effects of miR-34a on hepatocytes and thus how the affected hepatocytes influenced HSCs. Normal hepatocyte L-02 cells were transfected with miR-34a mimics (0, 20, 50 nM) and then treated with or without SRT1720. Upregulated concentration of miR-34a was observed in a dose-dependent manner in miR-34a mimic-transfected L-02 cells with or without SRT1720 (Fig 4A). SRT1720 treatment decreased the concentration of miR-34a a little while the difference was not statistically significant. This might because of the excessed miR-34a mimics in the transfected cells. SIRT1 expression decreased with the upregulation of miR-34a, and SRT1720 abolished the miR-34a-induced decrease (Fig 4B). Consequently, Ac-p53 was upregulated in miR-34a mimic-treated cells, and SRT1720 reversed the upregulation induced by miR-34a (Fig 4B).

The activation of the miR-34a/SIRT1/p53 signaling pathway was always related to apoptosis; thus, we examined the apoptosis of these treated L-02 cells. An Annexin V-FITC/PI kit was used to detect apoptosis. As shown in Fig 4C, miR-34 significantly induced the apoptosis of L-02 cells in a dose-dependent manner, and the induced apoptosis was then inhibited by SRT1720. Further experiment studying the activation of caspase3 showed that cleaved caspase3 was significantly increased in a dose-dependent manner, and the cleavage of caspase3 was inhibited by SRT1720 (Fig 4D).

**Fig 5:**
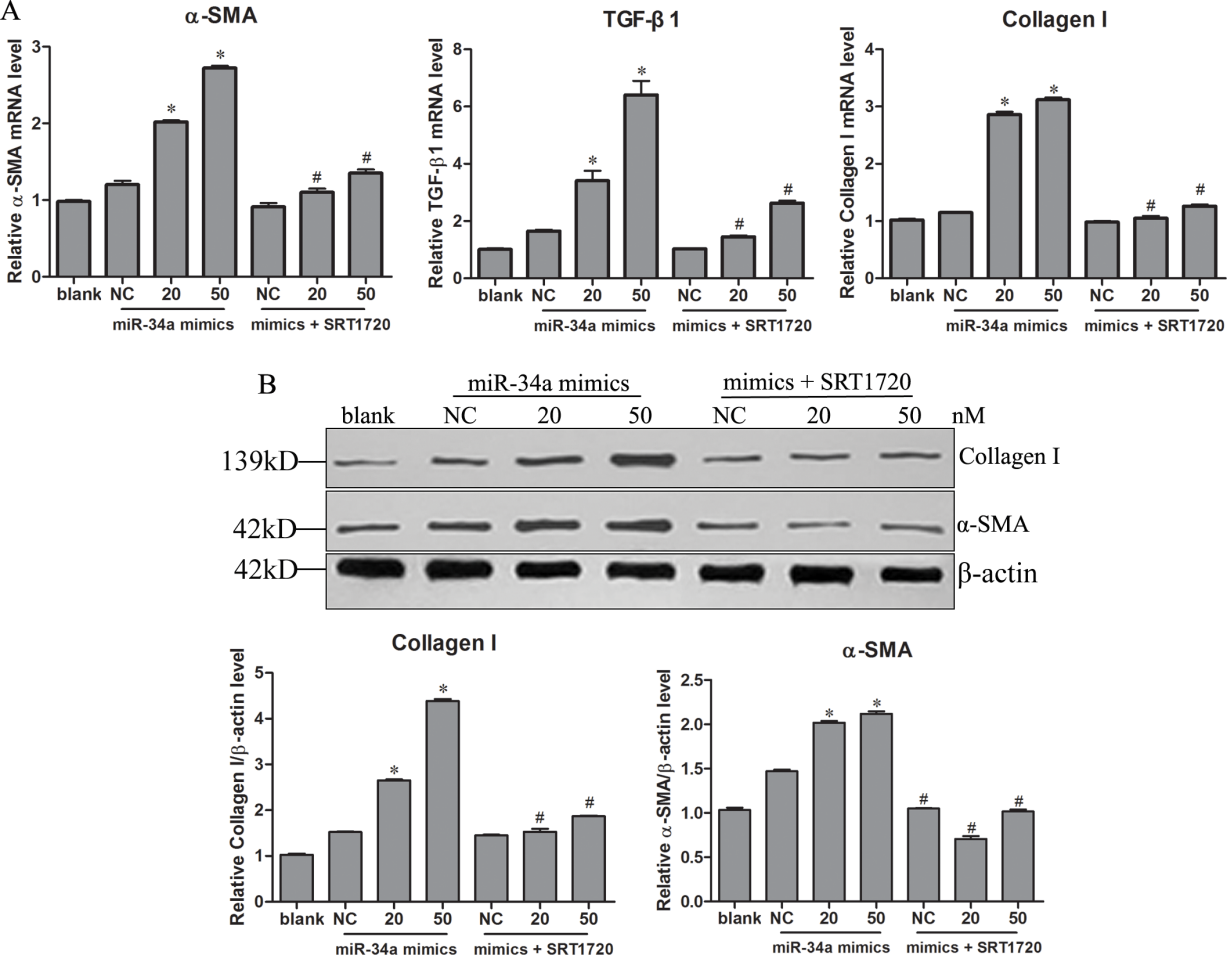
miR-34a-induced apoptosis of hepatocytes activates HSCs. HSCs LX-2 cells were co-cultured with pre-treated L-02 cells without SRT1720 for 24 h. L-02 cell were transfected with miR-34a mimics (20, 50 nM) in the presence/absence of SRT1720 for 24 h. The mRNA levels of α-SMA, TGF-β1 and collagen I (A) and the protein expression ofα-SMA and collagen I (B) in LX-2 cells were detected. * *P*<0.05 compared to NC and to miR-34a mimic-transfected cells. Blank, HSC LX-2 co-cultured with L -02 cells; NC, negative control (HSC LX-2 co-cultured with L -02 cells transfected with negative mimics). * *P*<0.05 compared to NC. # *P*<0.05 compared to mimics transfection.

Next, the effects of L-02 apoptosis on HSCs were examined. miR-34a mimic-transfected L-02 cells treated with or without SRT1720 were cultured for 24 h and then co-cultured with HSCs LX-2 cells without SRT1720 for another 24 h. The mRNA levels of several liver fibrosis-related proteins, including α-SMA, TGF-β1 and collagen I, and the protein expression of α-SMA and collagen I in HSCs were detected. The miR-34a-induced apoptosis of hepatocytes significantly increased the mRNA levels of α-SMA, TGF-β1, and collagen I (Fig 5A) and the protein expression of α-SMA and collagen I (Fig 5B). The increases in the mRNA levels and protein expression were significantly downregulated when miR-34a transfected hepatocytes were treated with SRT1720 (Fig 5A and 5B).

Taken together, upregulation of miR-34a induced the apoptosis of hepatocytes and thus activated HSCs through the activation of the miR-34a/SIRT1/p53 signaling pathway.

## Discussion

miR-34a is transcriptionally regulated by the p53 tumor suppressor protein and regulates a plethora of target proteins, which are involved in the cell cycle, apoptosis, differentiation and cellular development [25]. Downregulation of miR-34a has been found in many cancers [26–28]; thus, miR-34a is thought to be a tumor suppressor. However, previous studies have revealed that miR-34a is upregulated in many liver diseases, from fatty liver disease to HCC [29–31]. Upregulation of miR-34a may inhibit SIRT1, thus activating p53 and forming a positive feedback loop [22]. Although it has been reported that miR-34a can target acyl-CoA synthetase long-chain family member 1 and impair the lipid metabolism in the liver, resulting in the development of liver fibrosis [19], the function of the classical miR-34a/SIRT1/p53 signaling pathway in liver fibrosis is presently unclear. It was recently reported that the miR-34a/SIRT1/p53 signaling pathway was activated in NAFLD and participated in the apoptosis of hepatocytes [16]. We wondered if this classical miR-34a/SIRT1/p53 signaling pathway was also involved in liver fibrosis and how the pathway functioned during this process. In the present study, we investigated the functions of the miR-34a/SIRT1/p53 signaling pathway during liver fibrosis.

Using a CCl_4_-induced rat liver fibrosis model, the upregulation of miR-34a and Ac-p53 and downregulation of SIRT1 were observed. Moreover, activation of SIRT1 decreased the expression of miR-34a and the acylation of p53. These results revealed the activation of the miR-34a/SIRT1/p53 signaling pathway during liver fibrosis. Further experiments showed that the miR-34a/SIRT1/p53 signaling pathway was only activated in hepatocytes and not in HSCs. The activation of this pathway in hepatocytes resulted in the apoptosis of hepatocytes and thus activated HSCs.

Liver fibrosis is a complex pathological process, and the activation of HSCs is the pivotal event during the process [2]. However, HSCs occupy only approximately 5~8% of the total liver cells. As the major components of the liver, parenchymal hepatocytes are the major cells faced with different types of liver damage. Generally, after an acute liver injury, hepatocytes regenerate and replace the damaged apoptotic cells [32]. When the damage persists, the regenerative response eventually fails, and many hepatocytes undergo apoptosis [32]. The adjacent HSCs and other nonparenchymal cells phagocytize the apoptotic cells, thus resulting in the activation of HSCs and accelerating the liver fibrosis process [32, 33]. Our data indicated that the miR-34a/SIRT1/p53 signaling pathway participated in the liver fibrosis process by inducing the apoptosis of hepatocytes, and thus activating HSCs. This signaling pathway might be a potential therapy target for liver fibrosis.

As the central component of the miR-34a/SIRT1/p53 signaling pathway, SIRT1 plays an important role in liver fibrosis. Our data showed that stimulating SIRT1 expression significantly attenuated liver fibrosis, which was achieved through inhibiting hepatocyte apoptosis *via* decreasing p53 acylation and caspase activation. SIRT1 is a highly conserved NAD^+^-dependent protein deacetylase and a master regulator of the transcriptional networks controlling hepatic lipid metabolism [34, 35]. SIRT1 has long been identified as the target protein of miR-34a [36], although there have been no reports linking upregulation of miR-34a with SIRT1 in liver fibrosis. In fact, another widely used SIRT1 activator, resveratrol, has also been reported to attenuate liver fibrosis in animal models [37, 38]. The underlying mechanism, however, has not been fully elucidated. The miR-34a/SIRT1/p53 signaling pathway may contribute to the antifibrotic function of resveratrol.

Recently, the miR-34a/SIRT1/p53 signaling pathway was reported to be involved in human NASH and positively related to NAFLD severity, which is the result of a link between hepatocyte apoptosis and the miR-34a/SIRT1/p53 signaling pathway [16]. Our results also showed that activating the miR-34a/SIRT1/p53 signaling pathway induced hepatocyte apoptosis. Considering that upregulation of miR-34a is a common event in many liver diseases and that SIRT1 is frequently involved, we hypothesize that the activation of the miR-34a/SIRT1/p53 signaling pathway is closely related to hepatocytes and contributes to the progress of liver disease.

In conclusion, in this study, we found that the miR-34a/SIRT1/p53 signaling pathway was involved in liver fibrosis by inducing the apoptosis of hepatocytes but not HSCs. This signaling pathway, especially SIRT1, might be a promising therapeutic target for liver fibrosis.

## Supporting Information

S1 Text ARRIVE checklist. (PDF)
